# Understanding heterogeneity of human bone marrow plasma cell maturation and survival pathways by single-cell analyses

**DOI:** 10.1016/j.celrep.2023.112682

**Published:** 2023-06-24

**Authors:** Meixue Duan, Doan C. Nguyen, Chester J. Joyner, Celia L. Saney, Christopher M. Tipton, Joel Andrews, Sagar Lonial, Caroline Kim, Ian Hentenaar, Astrid Kosters, Eliver Ghosn, Annette Jackson, Stuart Knechtle, Stalinraja Maruthamuthu, Sindhu Chandran, Tom Martin, Raja Rajalingam, Flavio Vincenti, Cynthia Breeden, Ignacio Sanz, Greg Gibson, F. Eun-Hyung Lee

**Affiliations:** 1 School of Biological Sciences, Georgia Institute of Technology, Atlanta, GA, USA; 2 Department of Medicine, Division of Pulmonary, Allergy, Critical Care, and Sleep Medicine, Emory University, Atlanta, GA, USA; 3 Department of Medicine, Division of Rheumatology, Lowance Center for Human Immunology, Emory University, Atlanta, GA, USA; 4 Department of Hematology and Medical Oncology, Winship Cancer Institute, Emory University, Atlanta, GA, USA; 5 Departments of Immunology, Duke University, Durham, NC, USA; 6 Department of Surgery, Duke University, Durham, NC, USA; 7 Immunogenetics and Transplantation Laboratory, Department of Surgery, University of California San Francisco, San Francisco, CA, USA; 8 Department of Medicine, University of California San Francisco, San Francisco, CA, USA; 9 Division of Nephrology, Department of Medicine, University of California San Francisco, San Francisco, CA, USA; 10 Emory Transplant Center, Department of Surgery, School of Medicine, Emory University, Atlanta, GA, USA

**Keywords:** long-lived plasma cell, single-cell sequencing, heterogeneity and maturation, human bone marrow, TNF signaling through NFKB

## Abstract

Human bone marrow (BM) plasma cells are heterogeneous, ranging from newly arrived antibody-secreting cells (ASCs) to long-lived plasma cells (LLPCs). We provide single-cell transcriptional resolution of 17,347 BM ASCs from five healthy adults. Fifteen clusters are identified ranging from newly minted ASCs (cluster 1) expressing MKI67 and high major histocompatibility complex (MHC) class II that progress to late clusters 5–8 through intermediate clusters 2–4. Additional ASC clusters include the following: immunoglobulin (Ig) M predominant (likely of extra-follicular origin), interferon responsive, and high mitochondrial activity. Late ASCs are distinguished by G2M checkpoints, mammalian target of rapamycin (mTOR) signaling, distinct metabolic pathways, CD38 expression, utilization of tumor necrosis factor (TNF)-receptor superfamily members, and two distinct maturation pathways involving TNF signaling through nuclear factor κB (NF-κB). This study provides a single-cell atlas and molecular roadmap of LLPC maturation trajectories essential in the BM microniche. Altogether, understanding BM ASC heterogeneity in health and disease enables development of new strategies to enhance protective ASCs and to deplete pathogenic ones.

## Highlights

• Early BM ASCs contain clusters with high class I and II, IgM, IFN, and mitochondrial genes
• Late BM ASCs are distinguished by G2M checkpoints, mTOR signaling, and TNFRSF members
• Early ASCs have higher CD38 expression and are selectively depleted with anti-CD38 antibodies
• There is heterogeneity of late BM ASCs by degree of TNF signaling through NF-κB

## Graphical abstract

**Figure:**
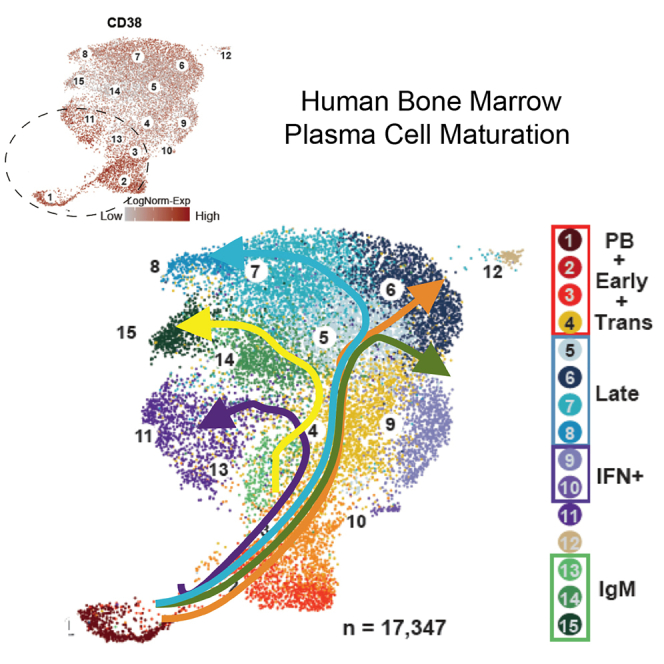

## Teaser

Although long-lived plasma cells (LLPCs) have been identified in human bone marrow (BM), the heterogeneity and mechanistic maturation trajectories have not been clearly defined. Duan et al. demonstrate the heterogeneity of the human BM plasma cells by providing a single-cell transcriptional atlas with a roadmap to LLPC maturation.

## Introduction

The existence of long-lived plasma cells (LLPCs) that provide a lifetime of humoral protection after vaccination and infection is well established in mice and humans. Activated lymph node B cells differentiate into early antibody-secreting cells (ASCs), which home to bone marrow (BM) niches where a fraction may survive as LLPCs, which we previously identified within the CD19^−^CD38^hi^CD138^+^ cell population.^1^^,^^2^^,^^3^^,^^4^^,^^5^^,^^6^ Whether mere migration to protective niches is sufficient for the establishment of an LLPC compartment or, instead, additional maturation in the BM is required was unclear. Thus, we recently showed that early maturation of nascent ASCs takes place in the BM through morphologic, transcriptomic, and epigenomic changes that presumably enable their ultimate differentiation into LLPCs.^6^ Accordingly, we postulated that peripheral ASCs arriving in the marrow undergo further maturation locally to generate *bona fide* LLPC.

We have also developed an *in vitro* human BM mimetic system, containing soluble factors from mesenchymal stromal cells (MSCs), a proliferation-inducing ligand (APRIL), and hypoxia, which sustains ASC survival for up to 56 days in culture, thereby overcoming previous experimental limitations in the field imposed by the rarity and *ex vivo* frailty of ASCs. This approach enabled a molecular roadmap charting the progression of nascent ASCs into mature ASCs demarcated by upregulation of CD138 expression and downregulation of CD19 as early as day 14.^7^ This early work showed that engagement of sequential transcriptomic and epigenetic programs promoting resistance to apoptosis is essential for survival.^6^ Although candidate LLPC BM maturation programs were revealed, bulk analyses could not directly interrogate the heterogeneity of the BM LLPC compartment.

Here, we have performed extensive single-cell analysis of BM ASCs. Our studies identify a large degree of cell heterogeneity and maturation trajectories. Starting from nascent KI67+ ASCs with the highest major histocompatibility complex (MHC) class II expression, we could assign early and late ASCs distinguished by differences in G2M check points, E2F targets, mammalian target of rapamycin (mTOR) signaling, and metabolic pathways for fatty acid and oxidative phosphorylation as well as differential expression of members of the tumor necrosis factor (TNF) receptor superfamily and CD38 expression. Finally, terminal differentiation of late immunoglobulin (Ig) G ASCs followed two distinct paths with differential utilization of the TNFα signaling pathway via nuclear factor κB (NF-κB). In all, this study provides a single-cell atlas and molecular roadmap trajectories of LLPC maturation in the human BM.

## Results

### Single-cell transcriptomic profiling of human plasma cells in the BM

**Figure 1:**
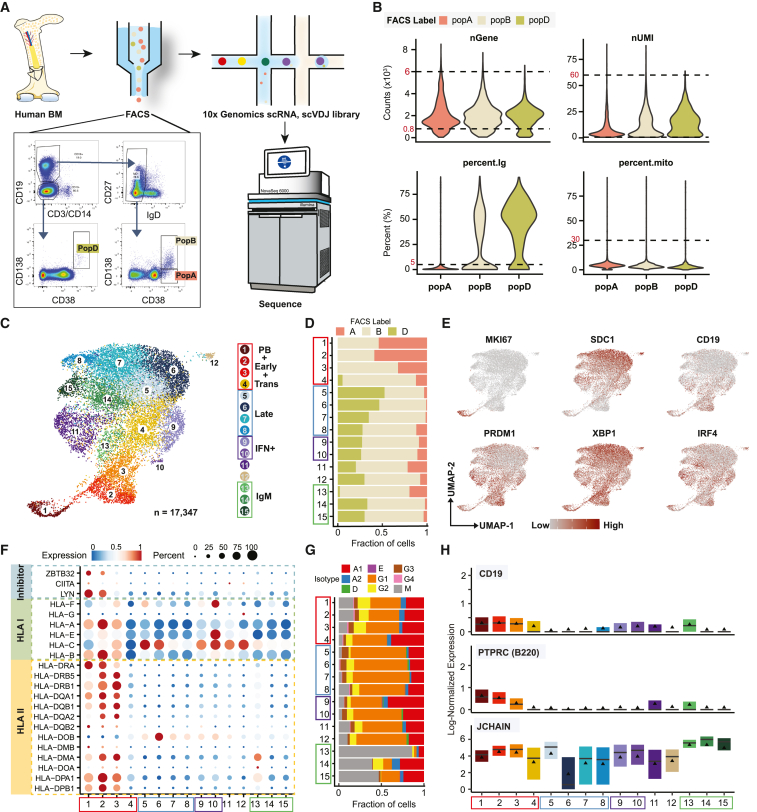
Single-cell transcriptomic profiling of bone marrow PCs (A) Schematic of single-cell RNA profiling for human BMPCs. (B) Criterion for removing bad-quality cells. Dashed lines show the cutoffs that are labeled in red. (C) scRNA-seq cell clusters of combined data from five healthy BMs and visualized in UMAP colored by cell types. Red and blue boxes highlight the early and late stage of BMPC maturation, respectively. The purple box highlights the path toward IFN-response PC subgroups and the green box highlights IgM-dominant cell populations. (D) The fraction of cells from fluorescence-activated cell sorting (FACS)-sorted cell population in each cell subgroup identified in (C). (E) Key PC-associated master gene expression. The redder the dot, the higher the log-normalized gene expression. (F) Dot plot for expression of human MHC class I, II, and inhibitors of class II genes. Colors represent minimum-maximum normalized mean expression of marker genes in each cell group, and sizes indicate the proportion of cells expressing marker genes. (G) The fraction of isotypes identified by scVDJ-seq data in each cell subgroup identified in (C). (H) Boxplots showing the expression levels of the indicated genes. The solid triangle represents the average value.

To characterize the heterogeneity of human bone marrow plasma cells (BMPCs), the three major populations previously identified were sorted from five healthy adults without recent immunization or infection^3^: pop A (CD19^+^CD38^hi^CD138^−^), pop B (CD19^+^CD38^hi^CD138^+^), and pop D (CD19^−^CD38^hi^CD138^+^) (Figure 1A). Although pop D constitutes the main reservoir of LLPC, to test the distinct molecular programs reflecting their generation, regulation, and survival, we evaluated the BM ASCs at the single-cell level using 5′-directed cDNA library construction to characterize both the single-cell transcriptomic profiles (single-cell RNA sequencing [scRNA-seq]) and matching single-cell V(D)J repertoire sequencing (scVDJ-seq). After exclusion of non-ASC contaminating cells (including B cells), low-quality cells, dying cells, and doublets, the remaining cells were identified as *bona fide* ASCs with the characteristic expression of *XBP1*, *IRF4*, *PRDM1*, *CD27*, *CD138*, and *CD19* (Figures 1B, 1C, 1E, S1C, and S2A). In total, we retained 17,347 ASCs.

#### Identification of 15 clusters of BM ASCs

Clustering was performed with the removal of known Ig genes and without knowledge of subsets A, B, and D *a priori*. However, our initial clustering required the elimination of two subsets falsely identified solely on the basis of very high expression of two misannotated Ig VH genes. Ultimately, we identified 15 robust clusters representing different cell states or types based on gene expression profiles (Figures 1C, 1E, and S2A–S2C). After establishing the 15 clusters, we re-incorporated the Ig genes and found no specific VH gene driving a particular cluster. These 15 distinct clusters (Figure 1C) could be grouped as early (clusters c1–3), transitional or intermediate (c4), and late (c5–8) ASC based on human leukocyte antigen (HLA) class I and II expression (Figure 1F). In addition, we identified two interferon (IFN)-response-dominated clusters (c9 and 10); and three IgM-predominant clusters (c13–15, Figure 1G) that contained the majority of BM IgM ASCs. Finally, we identified a mitochondrial-high cluster (c11) with characteristics of early ASCs and a minor cluster (c12), likely representing dying ASCs. The differentially expressed marker genes used to adjudicate each cluster are shown in Figure S2F and Table 1.

**Table 1:** Differentially expressed marker genes define the 15 clusters

Cluster ID	Cell label	Markers	Cluster ID	Cell label	Markers
1	PB	MKI67, UBE2C, BIRC5, SPN, TUBB	9	pc9	ATF5, PSAT1, CEBPB, ERN1
2	early 1	CD52, PEBP1, MHC class II (except HLA-DOB)	10	IFN+	MX1, XAF1, IRF7, STAT1
3	early 2	CD79A, CD74	11	Mito-high	MALAT1, IRF4, PRDM1, ZBTB20, MTRNR2L12, FCRL5
4	trans	IGLV3-25	12	pc12	JUND, CXCR4, SIK1B
5	late 1	SMOC1, MOXD1	13	IgM1	CCDC88A, FOXP1, IGHM, KLHL14, MS4A1
6	late 2	CD9, CST3, CD63, TIMP1	14	IgM2	JCHAIN, RGS2, TNFRSF4
7	late 3	RHOB, CDKN1A, RGCC	15	IgM3	CCL3, CCL4
8	late 4	JUNB, FOS, EGR1, NR4A1	–	–	–

#### Separation of early and late clusters by HLA class II gene expression

c1 distinctly expressed MKI67, CD43 (*SPN*), and CD27 and had low expression of CD20 (*MS4A1*) (Figures S2F and S2H). This pattern is consistent with the definition of proliferative plasmablasts (PB) or early-minted ASCs, representing new BM arrivals from active immune responses (CD20^low^CD27^high^CD43^high^).^8^ As they mature into resting LLPCs, proliferative ASCs typically shed B cell markers such as surface Ig and MHC class II genes (HLA gene complex).^3^^,^^4^ c2 and c3 lacked Ki67 expression but retained the highest levels of HLA class II genes, which subsequently decreased in the transitional c4 (Figure 1F). Thus, the initial ASCs seeding the BM could be further divided into early stages, including proliferative and non-proliferative cells (c1, and c2 and 3, respectively), and transitional stages (c4). Interestingly, c1–3 were devoid of pop D, which could be first detected at low frequencies within c4 (Figure 1D).

As expected, *PRDM1*, encoding BLIMP1, which drives plasma cell (PC) commitment and extinguishes class II transactivator *CIITA* and MHC class II gene expression, was expressed from the early ASC stages^9^ (Figure 1F). In turn, *CIITA* was universally extinguished on nearly all BM ASC subsets except for a small fraction of early c1. HLA-DOB, which suppresses peptide loading of class II molecules, was upregulated in the late ASC clusters, which is consistent with reports that HLA-DOB is less affected by *CIITA* than other family members.^10^ HLA class I expression followed similar trends except for HLA-C. In all, HLA class II expression provides clear separation of early and late BM ASC subsets.

#### Marker genes of human BM ASC clusters

We next identified cell-type-specific markers across the 15 BM ASC subgroups (Figure S2F; Table S1). In addition to the defining features, Ki-67 and HLA class II, other markers of early ASCs included *LYN* and *ZBTB32*. LYN is a tyrosine protein kinase downstream of BCR signaling that can diminish proliferation while driving terminal PC differentiation^11^ (Figure 1F). Interestingly, LYN deficiency leads to a 20-fold increase of BM PCs and induces autoimmune disease in mouse models.^12^ Thus, progressive extinction of LYN expression in late clusters may contribute to enhanced survival of the more mature PCs.^13^ Also informative for early maturation were B cell markers *CD19* and *PTPRC* (B220), which were strongly expressed in early IgG-dominant c1–c3 as well as in c11 and IgM-dominated c13 (Figures 1G and 1H). The progressive loss of *CD19* and *PTPRC* during B cell differentiation into PC and the ability of CD19^+^ B220^+^ cells to secrete low-affinity IgM antibodies^14^^,^^15^ suggests early populations.

The transitional nature of c4 was documented by the progressive loss of early features including MHC class II and initial expression of late markers. In turn, c5 shared with c4 higher levels of transcripts characteristic of late subsets, including *MDK*, *ITM2B*, *LMNA*, *AREG*, and *TIMP1* (Tables 1 and S1).

Late c6–c7, and to a lesser extent c8, expressed higher levels of *CD9* and *CST3*. The CD9 tetraspanin modifies multiple cellular events of relevance for BM ASCs, including adhesion, migration, proliferation, and survival. Although *CD9* expression has been considered to mark GC-derived human PCs,^16^ *CD9* is also considered a marker of mouse PCs derived from marginal zone and B1 B cells in primary T-dependent responses.^17^

Finally, genes involved in regulation of cell-cycle arrest, apoptosis, and survival were preferentially expressed in c7 (*CDKN1A*/p21 and *RGCC*) and c8 (*JUNB*, *FOS*, *EGR1*, *NR4A1*; Table 1). P21 is a potent cyclin-dependent kinase inhibitor whose expression regulates cell-cycle progression and is tightly controlled by the tumor suppressor p53, which mediates cell-cycle arrest and can promote apoptosis in a context-dependent fashion.^18^ Early Growth Response (*EGR1*) is a nuclear transcriptional regulator of multiple tumor suppressors, including p53. Notably, *EGR1* is rapidly induced by growth factors, apoptotic signals, and hypoxia, a feature of the BM microenvironment that determines ASC survival.^19^^,^^20^ Of note, it has been shown to play a non-redundant role in PC differentiation.^21^ While the induction of the orphan nuclear receptor *NR4A1* (Nur77) is best recognized as a consequence of antigen receptor engagement in B and T cells,^22^ *NR4A1* is also induced by other stimuli, including endoplasmic reticulum (ER) stress, which is present at high levels in PCs secondary to high Ig synthesis.^23^ Interestingly, *NR4A1* can modify the pro/anti-apoptotic balance of the Bcl2 family and has enhanced binding to anti-apoptotic Bcl-B, which is prominently expressed in PCs.^24^^,^^25^

Notably, c7 and c8 are separated from other late ASC populations (c5 and c6) by the highest levels of the TNFα-signaling NF-κB pathway. These marker genes, especially of the late clusters, provide essential clues to unique mechanisms that support survival.

IFN-responsive genes (IFN+) were the major markers in c9 and c10. C9 highly expressed *ATF5*, *CEBPB*, and *ERN1*, whereas c10 expressed the classical IFN-response signature (*STAT1*, *IRF7*, *ISG15*, *IFITM1*, *IFI6*, *MX1* and *OAS1*).

We identified all five isotypes (IgM, IgG, IgA, and a small fraction of IgD and IgE), as well as the four IgG subclasses (IgG1–IgG4), in BM ASCs (Figure 1G). The majority of ASC clusters were dominated by IgG, predominantly IgG1. Compared with early stages, late stages (c5–8, and c10–12) had expanded proportions of the IgG isotype (p <2.2e^−16^).

#### Mature IgM ASC populations in the human BM

C13–15 contained the largest repository of IgM cells (40%–80%) (Figure 1G), which represented a large majority of c13. Interestingly, these IgM-predominant clusters displayed an overall distinct transcriptome and could in turn be split into early (c13) and late clusters (c14 and 15), according to the HLA expression and scarcity of pop D cells in c13 (Figure 1D). However, it is likely the maturation programs of IgM versus IgG trajectories would follow different paths. Thus, the IgM clusters are candidates for the human counterpart to mouse IgM LLPCs, which accumulate in the spleen in a GC-independent fashion and contribute to protective IgM responses.^26^^,^^27^^,^^28^

IgM ASCs (c13–15) are further defined by higher expression of *CCR10*, *JCHAIN*, *FHL1*, *PHACTR1*, and *RAMP2* relative to IgG-dominant cell populations (Figures S3A and 1H). Since C-C motif receptor 10 (CCR10) and JCHAIN are widely expressed in mucosal ASCs,^29^^,^^30^ the IgM-dominant cell populations may have mucosal origins. Additional differentially expressed genes (DEGs) distinguishing IgM- from IgG-dominant clusters included 77 *CCR10* co-expressed genes summarized in Figure S3B. These *CCR10*-related genes included *EBI2* (*GPR183*), *JCHAIN*, *FOXP1*, and several genes involved in regulation of lymphocyte activation, such as *FCRL3*, *TNFSF9*, *CLECL1*, and *FGL2*. *FOXP1* is known to impair the formation of germinal centers (GCs) and to repress human PC differentiation but are highly expressed in mature primary human B cells (e.g., naive B, memory B cells) as well as in mouse follicular B and B-1 cells.^31^^,^^32^ This profile is consistent with a GC-independent extra-follicular origin of the late IgM ASCs.^32^^,^^33^

### Relationship of BM ASC single-cell clusters to previously described ASC populations

Consistent with previous models of BM ASC maturation,^3^ *CD19* expression was notable in early (c1–4) and extinguished in late clusters, while SDC1 (*CD138*) expression was highest in the late clusters (Figure 1E). *XBP1*, an essential transcription factor (TF) associated with the ASC unfolded protein response (UPR), was increased in all clusters. Contrary to mouse studies, *PRDM1* expression was higher in early subsets and did not continuously increase in all late subsets (Figures 1E and S4A), a finding noted by bulk transcriptomes and intracellular BLIMP1 staining.^3^ Thus, *PRDM1* upregulation was limited to a small fraction of late clusters c7, c8, and c15, indicating that the high expression of this essential PC TF may be required for early ASC differentiation yet less important for terminal differentiation or maturation and long-term survival.^3^^,^^34^^,^^35^

The majority of pop D was found in the late stages of ASC maturation and, Interestingly, it was distributed across c5–c8, which also included a minority of pop A cells but transcriptionally resembled pop B (Figures 1D and S4D). A smaller fraction of pop D was spread across late c5–c12 and c14–c15. In contrast, pop B contributed nearly half of the cells in each of the early and late clusters, again suggesting that it corresponds to an intermediary BM population.

Both these early (c1–4) and a “mito-high” subgroup (c11) had higher *CD19* and *PRDM1* expression, higher number of total detected genes, and lower percentage of Ig transcripts relative to late mature ASCs (Figures 1E and S4A–S4C). Combined with their higher expression of *IRF4*, *ZBTB20*, and *FCRL5* (Table 1), this cluster appears to belong with the early ASC populations.^36^

### Identification of four maturation paths for IgG BM ASCs through trajectory analysis

**Figure 2:**
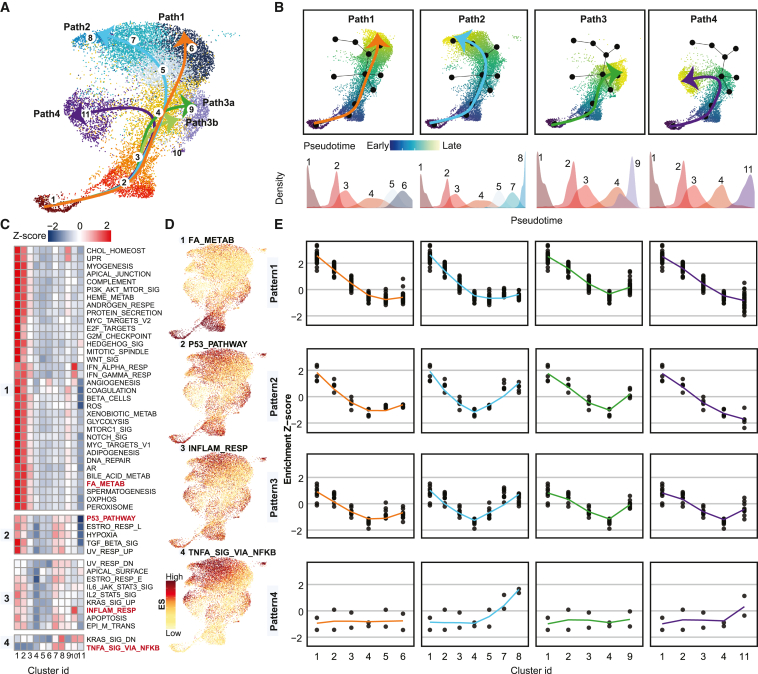
Trajectory and hallmark pathway enrichment analysis to distinguish predicted BMPC maturation paths (A) UMAP plot shows predicted paths of IgG1-dominant BMPC maturation. Arrows indicate the maturation direction. (B) UMAP plots show cell cluster located in each maturation path and colored by predicted pseudotime. (Top) Each solid black dot represents a cell population used to predict path in (A). The darker the blue and the lighter the green, the earlier and later stages of maturation, respectively. (Bottom) The density plots show the distribution of scRNA-seq-identified cluster on pseudotime space of each path. (C) Heatmap shows the row-scaled enrichment scores (ESs) for hallmark pathway enrichment analysis in the IgG1-dominant cell populations; from left to right is c1–c11. (D) Projected ES of indicated pathway example from each pattern onto UMAP. The darker the red, the higher the enrichment. (E) Dot plot next to the example UMAP visualization in (D) shows the scaled ES from each pattern by cluster from each path. The x axis is ordered cell populations corresponding to the cell order in each path in (B). Loess method fitted lines of ES alteration trends were colored by predicted paths in (B).

IgG ASCs comprise the major isotype in the late clusters; thus, we used Slingshot to construct maturation trajectories to focus on IgG BM ASCs and visualize the dynamic alteration of gene sets by pseudotime for each path onto the uniform manifold approximation and projection (UMAP) (Figures 2A and 2B). This approach predicted five maturation trajectories. Path 1 and 2 project to late c6 and c7 and 8 respectively. Path 3a and 3b, which directed to the same terminal c9, were merged into a single path 3. Finally, path 4 led toward c11. There was distinct separation between c1 and c2, but the remaining non-proliferative clusters in each path tend to be shared between adjacent stages/clusters. This result suggests the maturation of BM ASCs is more of a continuum of functional processes rather than an ordered sequence of discrete cell states.

Next, using hallmark pathway enrichment scores,^37^ we discovered four patterns describing the dynamic alteration of gene sets during BM ASC maturation (Figures 2C and S5A). From each pattern, a pathway example for visualization is shown in Figure 2D. Projecting scaled enrichment score (ES) from each pattern in Figure 2C onto pseudo-space paths inferred in Figure 2B, patterns 1, 2, and 3 exhibit a linear-like decrease in the pre-late stages but slightly different enrichment patterns in the late phase (Figure 2E). More specifically, pattern 1, which includes pathways of the UPR, reactive oxygen species (ROS), oxidative phosphorylation (Oxphos), and fatty acid metabolism (FA_METAB), decreases but then plateaus in late phase of maturation for paths 1, 2, and 3. Only path 4 decreases continuously into the late phase (Figure 2E). Pattern 2 includes the hallmark pathways hypoxia, UV response up, and p53 pathway, showing the same maturation trends but an increase in late states of path 2 corresponding to c7 and c8. Pattern 3 contains IL6-JAK-STAT3 signaling, inflammatory response, and apoptosis, revealing similar maturation trends in all four paths. Pattern 4, notably including TNFα signaling via NF-κB and KRAS signaling down, is most intriguing since the enrichment diverges between the late ASC in path 1 and 2. In addition to the IgG trajectories, we found high ES in IgM-pre-dominant lineage (c13–15) for TNFα signaling via NF-κB (Figure S5B) in c15 (Figure S5C).

#### TFs of ASC maturation and survival

**Figure 3:**
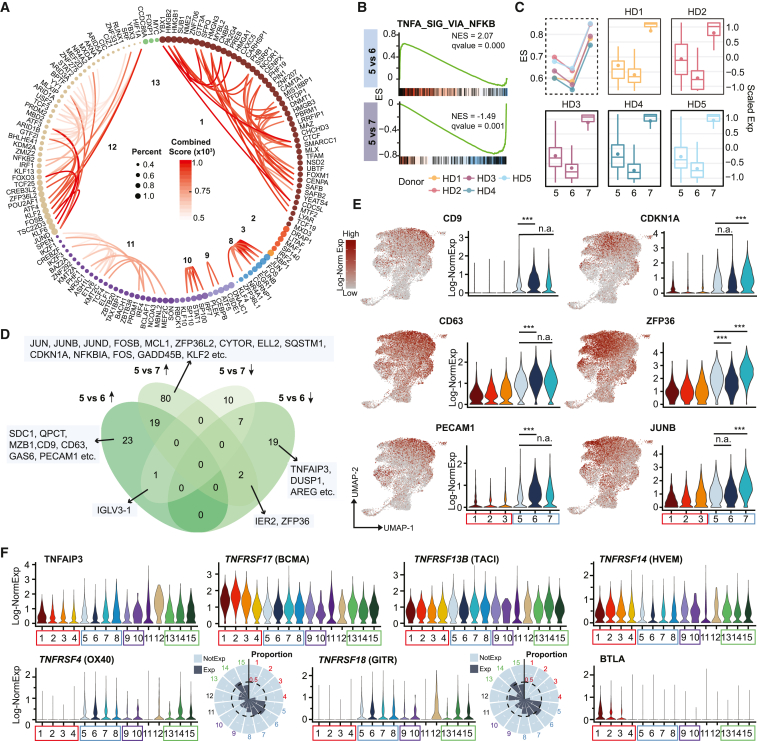
Genes and hallmark pathways distinguish late-phase maturation fate (A) Cell-cluster-associated TFs; each node represents a TF, colored by associated cell cluster and scaled by the expressed percentage of cells in the cluster. The lines between nodes are inferred protein-protein interactions from the STRING database. The redder the line, the more confident the inferred interactions. Numbers in the circles show the assigned cell cluster ID. (B) GSEA of the most indicated pathways. TNFα signaling via NF-κB that are differentially enriched between 5 vs. 6 and 5 vs. 7. (C) TNFα signaling via NF-κB hallmark pathway ES separated for each of the five individual subjects (in dashed box and y axis is on the left) and the distribution of scaled corresponding pathway enriched maturation-associated DEG expression in c5, c6, and c7 (in solid boxes and y axis is on the right). (D) Comparison of top DEGs between c5 vs. c6 and c5 vs. c7 (see star methods), based on the sign of average log fold-change (avglogFC), the DEGs were divided into up-/downregulated in c6 or c7 groups. Venn diagram shows the results of the DEG comparison. (E) Gene expression examples from the results of the comparisons in (D). (F) Gene expression of genes from TNF family. The x axis is cell cluster ID and y axis is the log-normalized gene expression. Circular bar plot shows the proportion of cells in each cell subgroup showing expression of corresponding genes. Black dashed line indicates the proportion of 0.5 and numbers indicate the cell cluster IDs. NotExp, not expressed; Exp, expressed. ^∗∗∗^Bonferroni-adjusted p value <0.001; n.a., not available.

To understand distinct enrichment patterns of gene sets regulated by specific TFs, we imputed the potential functions of TFs in regulating BM ASC maturation. We observed 205 differentially expressed TFs that are expressed in at least 10% of assigned cell clusters (Table S2). The most abundant 144 TFs were assigned to the cell cluster or defined stage of maturation (Figures 3A and S2C) with the highest gene expression. These “cluster-associated” TFs were observed in nine of 15 cell groups. Notably absent were any TFs defining the transitional and late-stage clusters c4–c7 or c14 and c15.

Adding protein-protein interaction information from the STRING database,^38^ we observed that some TFs are cluster specific (early or late) (Table S2). Thus, 48 out of 144 (33%) of the TFs were c1 associated and typically known to be involved in regulating basic functions such as RNA/DNA binding, transcription, metabolic processes, RNA stabilization (YBX1, SUB1), high-mobility group protein B (HMGB) damage-associated molecular patterns (DAMPs),^39^ (HMGB1, HMGB2, and HMGB3), and cell-cycle progression (MYBL2)^40^. Similarly, TFs important for immune activation, signaling, differentiation, and survival (EGR1, JUN, JUNB, FOS, NR4A1, and ZFP36L1) were in c8.

Two other large groups of 25 (∼17%) and 43 (∼30%) TFs were identified in c11 and 12, respectively. High mitochondrial gene expression in c11 suggests high stress and potentially eventual death. c12 was characterized by accumulation of ATF4, the first stable product that can trigger the activation of the PERK pathway, which promotes ASC apoptosis when ER stress is unabated.^41^^,^^42^ Thus, this small cluster may indeed be a death-prone ASC.

#### Bifurcation of LLPC differentiation

To characterize the bifurcation of late IgG ASC maturation in path 1 (c5 vs. c6) and path 2 (c5 vs. c7), we used gene set enrichment analysis (GSEA) prerank analysis and identified six and 19 gene sets that were significantly activated or repressed (Table S3). Although IFN-γ response and allograft rejection were downregulated in c6, inflammation and MYC targets v1 were upregulated in c7, and only TNFα signaling via NF-κB was downregulated in c6 and upregulated in c7 (false discovery rate ≤0.001) (Figure 3B). After evaluating the pathway ES for each individual separately, we found that this difference was not driven by any one subject (Figure 3C).

By comparing DEGs between c5 vs. c6 and c5 vs. c7, the Venn diagram shows top DEGs that are up or down in c6 or c7 (Figure 3D; Table S4). The expression of corresponding genes is visualized in Figure 3E. Subsequently, we compared the TNFα signaling via NF-κB directly between c6 and c7 and discovered 78 genes contributing to the significant enrichment (Figures S6A and S6B; Table S5). Out of those 78 leading edge genes, 34 (∼44%) are among the top DEGs, including *TNFAIP3*, *FOS*, *NFKBIA*, *CDKN1A*, and *ZFP36* (Figure S6C; Tables S4 and S5).

#### Differentially expressed TNFRSF family members between early and late ASC

TNF and TNF superfamily cytokine signaling play important roles in B cells and PC survival and function or cell death. The best known factors BAFF or APRIL (*TNFSF13*) and their recognized receptors BAFF receptor (BAFFR:*TNFRSF13C*), Transmembrane Activator and CAML-interactor (TACI:*TNFRSF13B*) and B cell maturation antigen (BCMA:*TNFRSF13A/17*) are essential for ASC survival.^43^^,^^44^ APRIL binds strongly to BCMA and moderately to TACI, whereas BAFF binds weakly to BCMA and strongly to TACI.^45^ Although BAFF has been suggested to be important in mouse BM ASCs,^46^ APRIL is the critical cytokine for ASC survival and maturation.^7^^,^^47^ Finally, APRIL and not BAFF can bind to heparin sulfate proteoglycans (HSPGs) such as CD138, which concentrates APRIL at the cell surface, thereby increasing its effectiveness.^48^

We found little difference in expression of the members of the TNFRSF family between the two late IgG paths1 and 2; however, there were major differences between the early and late clusters (Figures 3F, S7A, and S7B). BCMA showed high expression in early ASCs and was significantly downregulated in late ASC clusters, while TACI expression was significantly increased in late clusters, albeit to modest levels (Figures 3F and S7A; Table S6). Although TACI has been described in tonsil and BM ASCs,^49^ we found high expression of TACI in c14, a late IgM-predominant ASC cluster (Table S6). In mice, TACI expression is highest on mature innate-like B cells such as marginal zone and B-1 B cells, which is critical for T-independent responses^50^^,^^51^; however TACI may also have a role in late BM ASCs. Finally, healthy BM ASCs do not express APRIL or BAFF, showing the need for exogenous sources of these survival cytokines.

Other receptors in the TNFRSF considered to play roles in T cell activation were differentially regulated in early vs. late BM ASC clusters (Figure 3F). For example, OX40 (*TNFRSF4*) and GITR (*TNFRSF18*) were both significantly upregulated in the late ASC clusters. In contrast, HVEM (*TNFRSF14*) expression was substantially increased in the early c2, 3, 4, 9, and 13 (Figure S7A; Table S6). In all, the sequential TNFRSF programs in early and late ASCs provide important insights into BM maturation.

**Figure 4:**
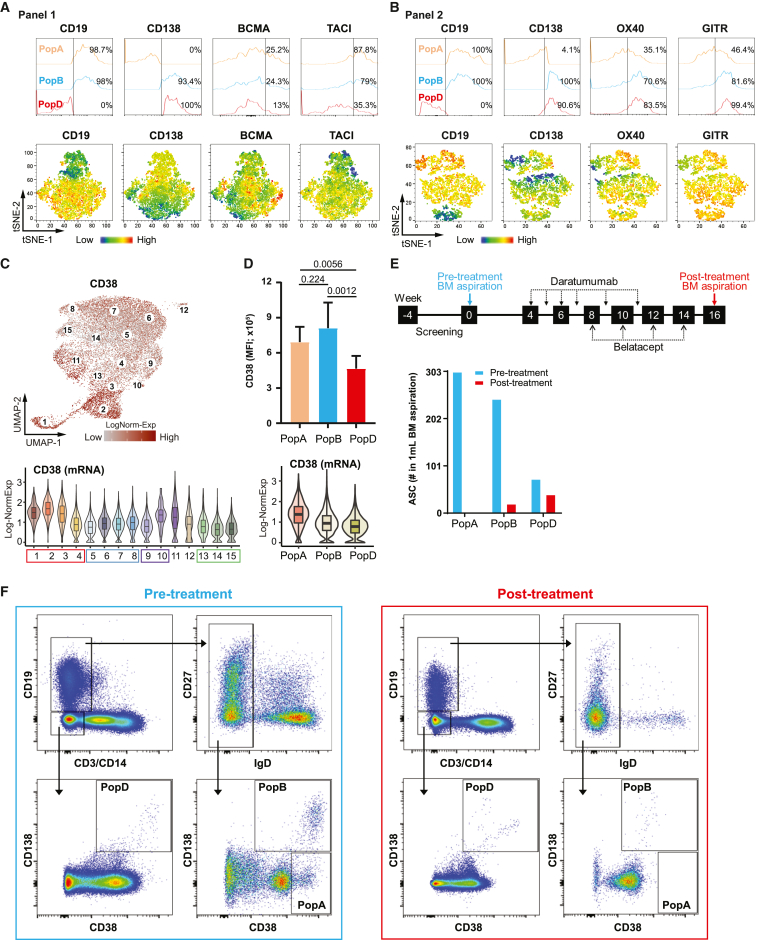
Experimental validation of early- and late-phase representative markers (A and B) Histograms (upper) and tSNE heat-maps (bottom) of expression of ASC surface markers (A) CD19, CD138, BCMA, and TACI (panel 1), or (B) CD19, CD138, OX40, and GITR (panel 2). (C) CD38 log-normalized gene expression visualized by UMAP (top) and violin plot grouped by cluster ID (bottom). (D) CD38 MFI measured from n = 9 healthy BM aspirates (top) and log-normalized gene expression visualized violin plot grouped by FACS-sorted cell labels (bottom). (E) Study regimen and timeline of the donor-specific antibodies (DSAs) (ClinicalTrials.gov identifier: NCT04827979, top). Quantitation of BM ASC subsets (# ASC/mL BM) pre and post treatment with daratumumab and belatacept (bottom). (F) Flow cytometry pre and post treatment.

Although protein expression may be concordant with gene expression,^52^ BCMA and TACI surface expression may be more variable and less concordant with gene expression (Figure 4A). Based on our previous BM pop A, B, and D, both BCMA and TACI decrease surface protein expression with maturation in pop D. In contrast, as BM ASCs mature, flow phenotyping revealed higher surface expression of OX40 and GITR in pop D, validating these expression profiles (Figures 3F and 4B).

Although CD38 expression increases during B cell differentiation to ASC precursors,^53^ its downregulation during ASC maturation was unanticipated; however, this feature may actually be consistent with enhanced survival in tumor cells.^54^ Pop D had lower mean fluorescence intensity (MFI) of CD38 by flow cytometry, which was concordant with gene expression (Figures 4C and 4D). Concordantly, we observed a selective loss of early BM subsets with anti-CD38 therapy (daratumumab). In a sensitized patient with a broad array of HLA antibodies awaiting the second kidney transplant, only the late mature ASC subsets remained after daratumumab and belatacept for 14 weeks (under an immune tolerance network [ITN] protocol ITN090ST; Figure 4E). We found depletion of pop A and B with 100% and 92.2% reduction respectively, while pop D remained (Figures 4E and 4F). Of the 59 HLA-specific serum antibodies pre-treatment, only 16 remained after treatment. Thus, early BM ASCs are the most susceptible to anti-CD38 therapies as predicted from the single-cell analysis. Combined, these data may help understand the therapeutic targeting achieved by anti-CD38 agents currently used for the treatment of PC malignancies and for highly sensitized patients and autoimmune conditions^55^^,^^56^^,^^57^^,^^58^ and finally provides an atlas and deep insights into the mechanistic implications of selective depletion of BM ASC subsets.

### Mature BM ASCs downregulate pro-apoptotic genes and upregulate pro-survival genes

**Figure 5:**
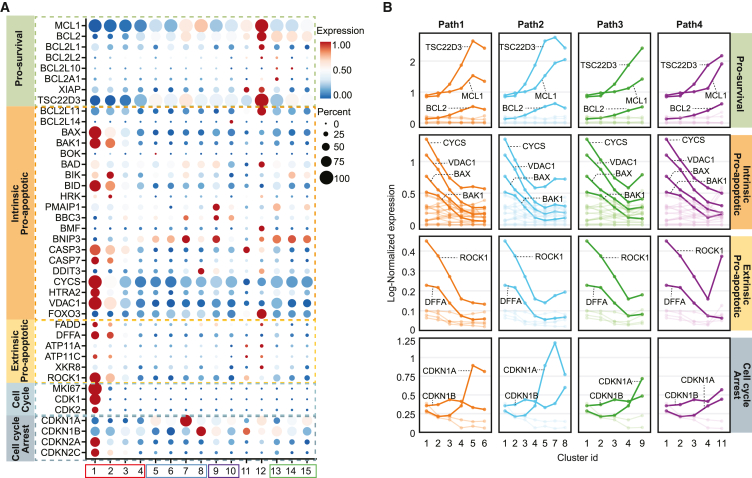
Exploration of apoptotic gene expressions in BMPC clusters (A) Dot plot showing the expression of genes related to pro-survival, intrinsic pro-apoptotic, extrinsic pro-apoptotic, cell-cycle progression, and cell-cycle arrest functions in BMPC subgroups. (B) Dynamic gene expression alterations in four maturation paths defined in Figure 2B with the same color coding and ordering. The labeled thick solid lines show the genes with high expressions and variations during the BMPC maturation.

Previous bulk RNA sequencing (RNA-seq) and assay for transposase-accessible chromatin with sequencing (ATAC-seq) indicated that pop D significantly upregulates pro-survival genes *BCL2* and *MCL1*, despite enhanced chromatin accessibility being present only for *BCL2*^6^ (Figure S7C). Here, we examined the single-cell expression of pro-survival, intrinsic and extrinsic pro-apoptotic genes, cell cycle, and cell-cycle arrest across the 15 clusters (Figure 5A). *TSC22D3*, *MCL1*, and *BCL2* are the essential pro-survival genes for BM ASC maturation. *TSC22D3* (glucocorticoid-induced leucine zipper [GILZ]) can inhibit the transcriptional activity of *FOXO3*, which leads to the further suppression of BIM-induced apoptosis, albeit in T cells.^59^ Of the late clusters, *TSC22D3* expression is high in c5 and c7 and the highest in c12, which has the corresponding highest expression of BIM (*BCL2L11*). *BCL2* is elevated to a similar degree in all late-stage LLPCs (c5–8), whereas *MCL1* shows even higher expression late in path 2 (c7 and 8; Figures 5A and 5B). Conversely, both intrinsic and extrinsic pro-apoptosis genes are reduced in the LLPC (path 1 and 2). These trends are consistent with a molecular basis for refractoriness to apoptosis in the late ASC.^60^

Inability to mature from early to late ASC likely leads to cell death (Figures 5A and 5B). c1 (PB) and c2 stages of BM ASC maturation appeared to be primed for apoptosis with higher expression of pro-apoptotic genes, including the mitochondrial outer membrane permeabilization (MOMP) activators *BAX* and *BAK1* and mediator VDAC1, and released apoptogenic proteins from the intermembrane of mitochondria cytochrome *c* (CYCS) and a serine protease OMI (encoded by *HTRA2*) that neutralizes the caspase-inhibitory proteins to directly and indirectly involve caspase activation. The DNA fragmentation factor (DFFA) and apoptotic membrane blebbing gene *ROCK1* were also upregulated.^61^^,^^62^^,^^63^^,^^64^ The PB stage also had a high abundance of transcripts important for proliferation, such as *MKI67*, *CDK1*, *CDK2*, as well as cell-cycle arrest-associated genes p16 (*CDKN2A*) and p18 (*CDKN2C*). Two other cell-cycle-inhibiting genes, p21 (*CDKN1A*) and p27 (*CDKN1B*), showed higher expression in c7 and c8, respectively.^65^ Cell-cycle arrest is largely regulated by activation of either one or both of the p16/PrB and p21/p53 pathways, which may be differentially regulated in early and late ASCs respectively.

c6 showed high expression of *CD138* (SDC1), a member of the heparan sulfate proteoglycan family which has been reported to promote ASC survival by regulating *BCL2* and *MCL1*.^66^ We also observed that *CD63*, *CD9*, and *IL5RA* were upregulated in the late-stage BM ASCs (Figure S7D). *CD9* and *CD63* have been reported to associate with the metastatic ability of tumor cells, such that higher expression promotes decreased cell motility.^67^ Interleukin (IL) 6 is known to be important for BM ASC survival,^7^^,^^68^ but, interestingly, IL6R expression was highest in the PB stage and c2, whereas IL6ST (binding of IL6 and IL6R) showed higher gene expression in c11 and 12 but not in the other late ASC clusters (Figure S7D). Hence, these results suggest that most BM ASCs likely utilize bound IL6 through the soluble IL6R.

### Isotype characteristics of BM ASCs

Our single-cell analysis provided a thorough representation of the whole V(D)J repertoire of human BM ASCs. Indeed, of the 17,347 cells with single-cell transcriptomic information, 83% also had matched VDJ:V_H_ (by scVDJ-seq) sequences including isotype identification. The V_H_ repertoire included 11,853 clonal lineages, with a majority representing singletons (10,344 cells), while 1,509 lineages contained at least two cells (Figure S8A). Of these, 421 were observed in only one of the 15 clusters, whereas 1,088 lineages were present in at least two cell clusters, mostly in adjacent UMAP spatial clusters (Figure S8B). Each cluster contained a similar percentage of singletons except c12, which contained few cells and the highest proportion of non-matched VDJ cells (Figures S8C and S8D). Nearly every cell (98.6% ± 0.8%) had a consensus Ig gene with greater than 100 transcripts (Figure S9), and single ASCs in the transitional and late stage had a higher proportion of cells with large numbers of Ig transcripts. The polyclonal repertoire was not unexpected since BM ASCs are the combined result of a lifetime of antigen exposures reflecting the historical serum antibody record.

### V(D)J repertoire characteristics of BM ASC clusters

**Figure 6:**
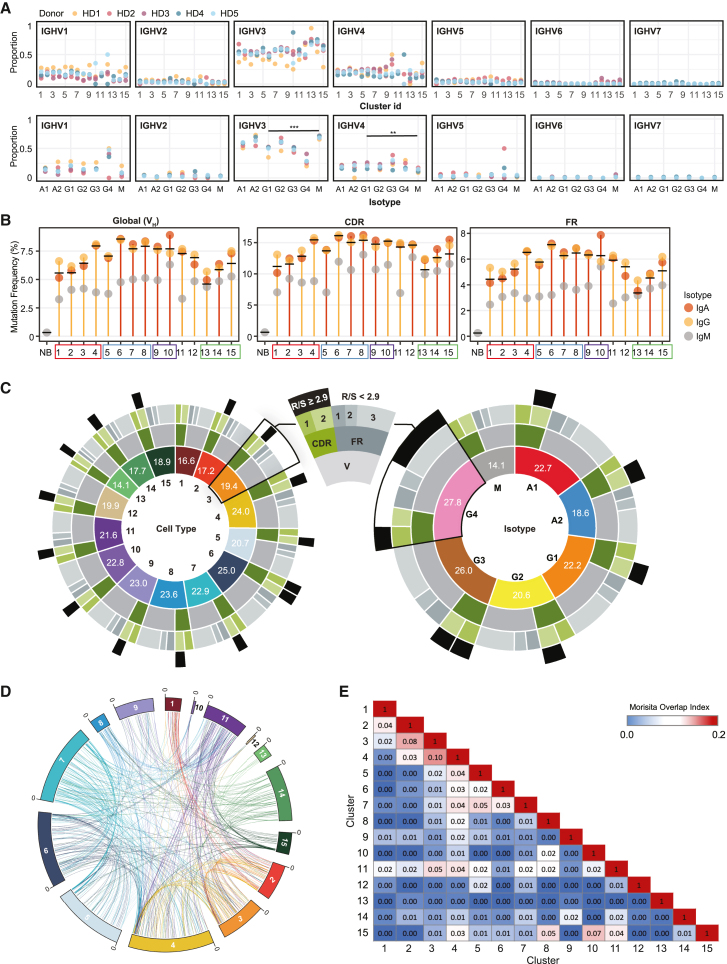
Mutation rate, similarity, and connectivity of clones measured by scVDJ-seq (A) Summary of Ig heavy-chain family gene usage. The y axis shows the proportion of cells from each cluster (top) and Ig isotype and IgG subclass (bottom). Black bar shows the comparison objects and asterisks indicate the significance of statistical test (^∗∗∗^p < 0.001, ^∗∗^p < 0.01). (B) The average mutation frequency in the whole region V (global), CDR, and FR of IgA, IgG, and IgM isotypes from each cell population. The solid black bar in each cell cluster indicates the overall average mutation frequency. NB, naive B cell as control. (C) Summary of the average mutation numbers by scRNA-seq-identified cell populations (left) and scVDJ-seq-identified isotypes (right). The innermost circle shows the cluster or isotype. Numbers in each section show the average number of mutations in region V. The second circle shows CDR and FR of region V. The third circle further breaks CDR and FR into CDR1, CDR2, FR1, FR2, and FR3. The outer-most circle black bar indicates the replacement-to-silence (R/S) ratio greater than 2.9. (D) Compiled Circos plot connecting individual subjects’ clones from cell c1 to c15. Lineages were colored by the latest cell cluster. (E) Heatmap showing the average Morisita overlap index of five subjects, with 0 indicating no similarity and 1 indicating identical repertoires.

V_H_ gene usage was not biased among ASC clusters, Ig isotypes, and individuals. Higher IGHV1, IGHV3, and IGHV4 family genes reflect the predicted distribution of these larger families (Figure 6A). Similarly, there was no consistent bias of individual VH genes within the VH families.

The frequency and distribution of somatic hypermutation (SHM) provides important information regarding the maturation and antigenic selection of ASCs and, by extension, may offer important insight into their differentiation from separate B cell sources. As expected, BM ASCs had higher global mutation frequencies relative to peripheral blood naive B (NB) cells (Figure 6B; NB = 0.33%, BM ASC = 6.89% ± 1.11%, p = 8.512e^−13^). The BM ASCs also displayed SHM frequencies comparable with peripheral memory B cells (7.1%).^69^

Overall, SHM frequencies ranged from 4.8% to 8.6%, with the lowest frequency observed in c13 and the highest in c6 (Figure 6B). IgG and IgA generally had significantly higher mutation frequencies than IgM isotypes, but there was not a difference between IgA and IgG isotypes or IgG subclasses within each identified cell cluster (Figure 6B; Table S7). Interestingly, the highest SHM mutation number was found in the small IgG4 fraction across clusters at 27.8 on average (Figure 6C). Early stage ASCs of the IgG lineage, especially those with MHC class II gene expression (c1–c3) as well as all IgM lineage clusters, had a lower mutation frequency than late-stage IgG ASCs. This result was evident across the global V region or within the framework region (FR) and complementarity-determining region (CDR), whether considered in isolation or combined (Figure 6B). There was a trend toward higher average mutation rates as BM ASCs mature, suggesting either a potential survival advantage of cells with higher mutation frequencies or, alternatively, preferential origination from late GC reactions. c6, which had the highest average number of mutations, also had the highest global V_H_, CDR, and FR region mutation frequencies (permutation test, p < 0.05; Figures S10A–S10D).

A replacement-silent (R/S) mutation ratio greater than 2.9 is suggestive of antigen selection^70^^,^^71^ and most of the clusters (14 out of 15) had a CDR2 R/S > 2.9. Only c6 showed an R/S > 2.9 in both CDR1 and CDR2, while c1 had no regions with R/S > 2.9 (Figure 6C). Overall, VDJ analysis showed that most BM ASCs are highly mutated and antigen selected.

#### Repertoire connectivity between early- and late-stage BM ASCs

Among the five individuals, we identified 11,853 V_H_ lineages, and all shared lineages were within a single individual, demonstrating no public clonotypes. Clonal lineages were defined as the same V, J, CDR3 length, and 85% CDR3 homology. Of the 11,853 V_H_ lineages identified, 1,088 (9.2%), were present in at least two ASC clusters, thereby indicating a significant level of cluster interrelationship (Figure S11B). However, to track the identical clone, we used 98% homology to follow the same clone in the pseudotrajectories. Since IgG1 ASCs are the dominant isotype in the BM, we show the connectivity of identical clones of IgG1 ASCs in c1–c15 in aggregate and by individual subject (Figures 6D and S11A).

We applied the Morisita overlap index to map lineage connectivity across populations. Cell populations were highly connected within the early (c1–c4) or the late stage (c5–c8) (Figures 6D and 6E). The degree of connectivity was substantially greater after removal of singletons, a strategy that enriches for larger clones and increases the sensitivity of detection (Figures S11B and S11C). Nearly identical clonal connectivity of IgG1 ASC in c1–c8 are shown (Figure S12).

Although some identical clones were detected in multiple cell clusters, one of the biggest, lineage #334, contained 11 cells distributed across early and late phases of IgG ASCs as well as c9, c10, c11, and c15 (Figure S13A). The VDJ sequences in this clone were identical, as shown in the alignment (Figure S13B). There were several clones with eight cells with identical sequences (385 and 280) that progressed through the early to late phases, validating potential paths for ASC maturation (Figures S13A and S13B). While these observations show representative clones, many other inter-cluster clones contained identical sequences as well. These observations are consistent with a dynamic BM ASC compartment composed of clones that likely undergo further maturation in the BM microniche.

## Discussion

ASCs represent a highly specialized effector compartment whose main physiological function is the production of protective antibodies, which, in some cases, may persist for the lifespan of the host, owing to the existence of LLPCs. While active infections trigger antibody production through newly made ASCs, either from naive or pre-existing memory B cells, long-standing antibodies represent a person’s immune history and contribute serological memory capable of preventing infection by previous agents. In contrast to other immune effector cells that perform their function during acute activation and are short lived, LLPCs are unique in that they constitutively perform their effector function while in a resting state and endowed with prolonged survival. In contrast, short-term ASCs, referred to as plasmablasts, perform the same effector function and may follow two different fates: post-proliferative apoptosis or subsequent maturation into LLPCs. It is well established that LLPCs persist in specialized survival microniches in the BM. However, it has remained uncertain whether they arrive in the BM as fully differentiated cells that only require admission to the protective niche or, instead, begin their BM journey from immature to mature PCs. In the latter scenario, it remains to be determined whether all new ASC arrivals to the BM represent potential LLPC precursors, or LLPCs derive only from specific ASC subsets. In either situation, critical outstanding questions include the specific phenotype of LLPCs among mature BM ASCs and the regulatory and survival trajectories of BM ASCs into LLPCs.

Our single-cell studies provide an in-depth analysis of human BM ASCs, thereby contributing substantial original insight into these questions. We provide a high-resolution atlas of the BM ASC, including the heterogeneity of the LLPC compartment. For example, this study offered mechanisms of known BM PC therapies (anti-CD38) together with discovery of novel therapeutic targets for mature BM plasma, such as GITR, OX40, CD9, CD63, ZBTB20, and IL5RA.

A finding of central significance is the identification of IgM BM ASC clusters with heterogeneous properties. While long-term IgM memory B cells are well established,^72^ the ability to generate mature IgM LLPCs has not been explored despite descriptions in the mouse spleen demonstrating persistent immunity.^28^ Combined, trajectory studies of clonal connectivity across these clusters show the presence of mature IgM BM PCs that might contribute long-lasting serum IgM antibodies. Future studies will be required to determine the precise contribution of extra-follicular (EF) and GC-driven pathways.^28^^,^^73^^,^^74^^,^^75^

Once they arrive in the BM, ASCs follow two major differentiation trajectories leading to mature PCs. These two paths offer differences of TNF signaling through NF-κB. AP-1 factors are also of particular importance among upregulated DEGs in path 2 (*JUN*, *JUNB*, *JUND*, *FOS*, *FOSB*, *MCL1*, *ZFP36L2*, *ELL2*, *CDKN1A*, *PRDM1*, *NFKBIA*, and others). Interestingly, JUNB is involved in proliferation and survival^76^ in diseased PCs (multiple myeloma [MM]) but is abundantly expressed in healthy ASCs, where it likely plays an anti-apoptotic role. Similarly, in MM, c-JUN is important for caspase-mediated c-ABL cleavage inducing apoptosis but may function quite differently in healthy BM ASCs. Whether the NF-κB/AP-1/STAT3 inflammatory regulatory network described in the transformation of human cancers^77^ also plays a role in LLPCs will require further study.

Trajectory analysis also points to an important role for cell-cycle regulation in late progression to terminally differentiated PCs through path 2. For example, p21, a marker capable of inhibiting a range of CDKs, is activated in a p53-dependent manner and was upregulated in c7 to mediate cell-cycle arrest.^78^ In turn, the tumor repressor p16 has also been described as a hallmark of cellular senescence.^79^^,^^80^^,^^81^ Exit from cell cycle irreversibly^82^ is a feature of cellular senescence and potential mechanism of LLPC survival. Path 2 was also characterized by higher expression of ZFP36L1, an RNA-binding protein (RBP), facilitates newly formed ASCs homing to the BM microniche.^83^ Although a role of RBP in ASC differentiation is unclear, these proteins may actually modulate ASC migration and survival in late ASCs.

Our study also provides insight into the metabolic and survival underpinnings of late-stage BM ASCs. Of particular interest for the latter property is the differential expression of receptors belonging to the TNFRSF family, specifically BCMA, which binds APRIL to enhance survival through MCL-1 in mice and to prolong survival in human LLPCs *in vitro* BM mimetic systems.^44^^,^^47^ Interestingly, while BCMA was expressed across all BM ASCs, it decreased 2-fold in more mature ASCs, thereby pointing to an important role in securing survival in early maturation. In contrast, TACI transcription experienced a modest increase from early to late BM ASCs, a finding consistent with its heterogeneous expression in MM^84^ and indicative of an unrecognized role in physiological LLPC maintenance. Although TACI is important for mouse ASC survival,^85^ its actual role and degree of redundancy with BCMA remain to be understood in humans. Finally, cell-cell contact through OX40 and GITR, which are only expressed in late ASCs, intimates a model where LLPCs may have limited motility due to cell-cell contact, as suggested in recent mouse studies.^86^

In our highly sensitized patients treated with daratumumab and belatacept, daratumumab likely resulted in the specific loss of early BM ASC subsets; however, we cannot totally rule out the role of belatacept in inhibiting newly generated ASC immigrants into the BM site. Despite these caveats, belatacept likely inhibited only a small proportion of BM ASCs within a 16-week period. Clearly, additional studies with single agents will be needed.

Our study offers major advances to the field of LLPC biology. First, we optimized isolation and viability of rare human PCs.^7^ Second, our experimental and analytical design achieved mRNA sequencing of sufficient depth for quantitative measurement of non-Ig genes, a goal readily compromised by the abundance of Ig transcripts representing up to 90% of some BM ASC total transcripts. Combined, our findings demonstrate substantial heterogeneity of mature BM ASCs and suggest that the LLPC potential may be present in several BM compartments. This possibility needs to be formally tested by localization of antigen-specific ASCs to the different clusters as previously done by our groups with the broader ASC populations. This goal could also be accomplished through the transcriptomes of identifying single ASCs of predetermined specificity.

### Limitations of the study

Limitations of this study include the small number of healthy adult subjects and the total number of *bona fide* PCs used in the analysis. However, it is good to have a representation of all the known human BM ASC subsets since we captured the rare CD19-CD138+ cells. Another limitation is the nature of the single BM aspirate that offers a one-time snapshot, while repeat sampling would afford longitudinal temporal trajectories. While providing valuable transcriptional information, this study does not provide epigenetic and functional activity of metabolic pathways.

## STAR★Methods

### Key resources table

**Table:** 

REAGENT or RESOURCE	SOURCE	IDENTIFIER
**Antibodies**		

IgD–FITC	BD Biosciences	Cat. No. 555778
CD3-BV711	BioLegend	Cat. No. 317328
CD14-BV711	BioLegend	Cat. No. 301838
CD19-PE-Cy7	BD Biosciences	Cat. No. 560911
CD38-V450	BD Biosciences	Cat. No. 561378
CD138-APC	Miltenyi Biotech	Cat. No. 130-117-395
CD27-APC-e780	eBiosciences	Cat. No. 5016160
LiveDead	Invitrogen	Cat. No. L34966
IgD-Brilliant Violet 480	BD Biosciences	Cat. No. 566138
CD3-BUV737	BD Biosciences	Cat. No. 612750
CD14-BUV737	BD Biosciences	Cat. No. 612763
CD19-Spark NIR 685	BioLegend	Cat. No. 302270
CD38-Brilliant Violet 785	BioLegend	Cat. No. 303530
CD138-APC-R700	BD Biosciences	Cat. No. 566050
CD27-Brilliant Violet 711	BioLegend	Cat. No. 356430
BCMA-Brilliant Violet 421	BioLegend	Cat. No. 357520
TACI-PE-Cy7	BioLegend	Cat. No. 311908
OX40-Brilliant Violet 510	BioLegend	Cat. No. 350026
GITR-Brilliant Violet 605	BD Biosciences	Cat. No. 747664
Zombie NIR Fixable Viability Kit	BioLegend	Cat. No. 423106
IgD-BB700	BD Biosciences	Cat. No. 566538
CD3-PE-Cy5	ThermoFisher	Cat. No. 2363822
CD14-PE-Cy5	ThermoFisher	Cat. No. 2319032
CD19-BUV395	BD Biosciences	Cat. No. 740287
CD38-FITC	Cytognos	Cat. No. CYT-38F2-A
CD138-PE-Cy7	BioLegend	Cat. No. 356514
CD27-BV605	BD Biosciences	Cat. No. 562655
Live/Dead	ThermoFisher	Cat. No. L34962

**Biological Samples**		

Bone marrow aspirate samples from a total of 11 healthy donors and 1 patient with high donor-specific antibodies awaiting kidney transplant.	Emory University and the ITN protocol (ClinicalTrials.gov Identifier: NCT04827979).	This paper.

**Critical Commercial Assays**		

EasySep cell isolation kit	StemCell	Custom-designed
Zombie NIR Fixable Viability Kit	BioLegend	Cat. No. 423106
Chromium Single Cell Human BCR Amplification kit	10X Genomics	Cat. No. PN-1000253
Chromium Next GEM Single Cell 5′ Reagent Kit, v1	10X Genomics	Cat. No. PN-1000165

**Deposited Data**		

scRNA-seq, scVDJ-seq raw and processed data	This paper	GSE230705
Original code	This paper	https://doi.org/10.5281/zenodo.7903579

**Software and Algorithms**		

Cellranger 3.0.1	10X Genomics	https://support.10xgenomics.com/single-cell-gene-expression/software/downloads/3.0/
Seurat 3.2.2	Butler et al.^87^	http://satijalab.org/seurat
SCTransform	Stuart et al.^88^	https://github.com/satijalab/sctransform
Slingshot	Street et al^89^	https://bioconductor.org/packages/release/bioc/html/slingshot.html
MAST	Finak et al.^90^	https://github.com/RGLab/MAST
GSEA v4.0.3	Subramanian et al.^91^	https://www.gsea-msigdb.org/gsea/index.jsp
UMAP	Becht et al.^92^	https://github.com/lmcinnes/umap
Molecular Signatures Database	Liberzon et al.^37^	http://software.broadinstitute.org/gsea/msigdb/index.jsp
STRING protein-protein interaction	Szklarczyk et al.^38^	https://string-db.org/

### Resource availability

#### Lead contact

Further information and requests for resources and reagents should be directed to and will be fulfilled by the lead contact, F. Eun-Hyung Lee (f.e.lee@emory.edu).

#### Materials availability

This study did not generate new reagents. Commercially available reagents are listed in the key resources table.

### Experimental model and study participant details

For the single cell BM data, 5 healthy adults were enrolled at Emory University Healthy in 2018 between the ages of 25 ± 4 years. All were female. An additional six healthy adults were enrolled at Emory University between 2019 and 2022 for flow cytometry validation. The mean age was 28.5 ± 6.4 years old and five were female. One male adult age 60 years old was enrolled at University of California San Francisco (UCSF) in the ITN ATTAIN trial who was highly sensitized. All studies were approved by the Institutional Review Board at Emory University and informed consent was provided.

### Method details

#### Cell sorting and library construction

Bone marrow aspirate was obtained under sterile conditions from the iliac crest from each of the 5 healthy adults. Mononuclear cells were isolated by Ficoll density gradient centrifugation and enriched by a custom-designed negative selection EasySep cell isolation kit from StemCell that removes CD66b+/GPA+/CD3+/CD14+ cells to limit sorting time of fragile BM ASC. Enriched mononuclear cells were stained with the following anti-human antibodies: IgD–FITC (Cat. no. 555778; BD Biosciences), CD3-BV711 (Cat. no. 317328; BioLegend), CD14-BV711 (Cat. no. 301838; BioLegend), CD19-PE-Cy7 (Cat. no. 560911; BD Biosciences), CD38-V450 (Cat. no. 561378; BD Biosciences), CD138-APC (Cat. no. 130-117-395; Miltenyi Biotech), CD27-APC-e780 (Cat. no. 5016160; eBiosciences), and LiveDead (L34966; Invitrogen). ASC subsets were FACS-sorted on a BD FACSAria II using a standardized sorting procedure with rainbow calibration particles to ensure consistency of sorts between individuals. ASC subsets were sorted as: popA (CD19^+^CD38^hi^CD138^-^), popB (CD19^+^CD38^hi^CD138^+^) and popD (CD19^−^CD38^hi^CD138^-^). Up to 17,000 cells were FACS-sorted from each population into RPMI with 5% FBS to maintain viability.

FACS-sorted cells were kept on ice until proceeding with 10x Genomics processing. Cells were centrifuged at 500xg for 10 min at 4C to remove media. Cells were then washed twice in 0.04% BSA in PBS. During last wash, media was aspirated and cell volume was measured using a pipette. The whole sample was then taken for processing using the 10x Genomics 5′ v1 Single Cell platform. V(D)J and 5′ Gene Expression (GEX) libraries were constructed for each sample, following 10x Genomics protocol. QC for each library was performed at each step by Bioanalyzer. Final libraries were quantified by kappa qPCR and sequenced on a NovaSeq.

### Quantification and statistical analysis

#### Pre-processing of 10x genomics scRNA-seq data and quality control

The 10x Genomics single-cell transcriptomic sequenced raw reads were aligned to GRCh38 reference and quantified per cell barcode using Cellranger v3.0.1 (https://support.10xgenomics.com/single-cell-gene-expression/software/downloads/latest). Genes expressed in at least 0.1%–0.3% (depending on the sample size) of the total cell population were regarded as expressed genes and retained for downstream analysis. To filter low-quality cells, we excluded cells with ≥ 30% of their UMIs coming from mitochondrial genes, or ≤ 800 total number of detected genes, or total number of UMIs ≤ 1000. We first retained cells with a detected gene number between 200 and 800 (labeled as LowGN in Figure S1A), but none of these genes were significantly expressed in the cell group. Moreover, we removed cells with total number of genes or UMIs ≥ 6,000 or 60,000, respectively, to control for potential doublets. Additionally, we filtered out cells with immunoglobulin genes corresponding UMI count percentage ≤ 5% to avoid contaminated non-antibody secreting cells. Then, we excluded contaminated cells expressing diagnostic cell markers, eg. CD3E (T cell), CD16 (encoded by *FCGR3A*) and CD14 (Monocytes), NKG7, GNLY (Natural killer cells), HBB (Erythrocytes), CD20 (encoded by *MS4A1*), PAX5, IRF8 (B cells) (Figure S1B).

#### Normalization and cell cluster detection

The scRNA-seq data was next analyzed using version 3.2.2 of the Seurat package.^87^^,^^88^ The gene expression counts of each cell were normalized using regularized negative binomial regression (SCTransform) to account for sequencing depth.^93^ Next, we selected the top 3,000 highly variable features (HVFs) based on their standardized expected variance after variance-stabilizing transformation, removed all the immunoglobulin genes from the HVF list, and then used HVF data from SCTransform residuals to perform principal component analysis (PCA). In the first run of our scRNA-seq analysis pipeline, we falsely included 2 misannotated Ig genes AC233755.1 and AC233755.2 in the HVF list, which resulted in 15 clusters plus 2 additional AC gene-driven cell clusters (Figure S2B). Subsequently, we further excluded these two genes before cell clusters detection. Next, we utilized Canonical Correlation Analysis (CCA) from r package Seurat to anchor each dataset, removing individual effect and generating an integrated dataset.^87^ Using this CCA-integrated dataset, a graph-based clustering method was applied to build a shared nearest neighbor (SNN) graph in PCA space, after which the Louvain community identification algorithm was applied to group cells at the set resolutions, with higher values leading to a greater number of clusters.^94^To assess the stability of the clustering based on different combinations of running parameters (dimension numbers = 50, 60 and 70, PC numbers = 30, 50, and 70 and resolution parameter = 0.2, 0.5, 1.0 and 1.5), we computed the Rand Index (RI) between pairs of classifications derived with different parameters. Accordingly, Rand Index (RI),^95^ which calculates the concordance of pairwise relationships between all pairs of cells, typically results in 86.5% similarity on average in cluster designation for each individual cell (Figure S2D).RI is a similarity measurement taking values from 0 (low) to 1 (high), which computes the proportion of cell pairs that are in agreement between cell clusters from two different parameters. Finally, we used 70 dimensions to anchor individual datasets and account for subject differences, and 50 PC were used to construct an SNN graph to detect cell clusters.^88^^,^^96^ A resolution of 1.0 gave the most stable cell clusters (Figure S2D). 15 cell clusters from the first run were retained with the cells from the two AC gene-driven clusters dispersed throughout the remaining 15 clusters. Finally, these clusters were visualized in two dimensions using uniform manifold approximation and projection (UMAP).^92^ Notably, the 15 subgroups were detected in all 5 individuals, with no identifiable subject-specific variability or Ig driven clusters (Figures S2C, S2E and S2G).

#### Differentially expressed and marker gene detection

Differentially expressed genes (DEG) between two cell clusters were identified based on LogNormalize SCTransform-adjusted count matrix, which is from data slot of SCTransform function in the Seurat r package. It’s worth noting that the HVF list with excluded Ig genes was used to detect cell clusters, the whole gene expression list which included Ig genes was used to detect cell markers and DEGs. It was performed by using zero-inflated generalized linear models including individual as a random covariate, with the MAST package,^90^ which was proven effectiveness on both real measured and simulated single-cell data.^97^ Cell cluster marker gene detection used the same settings but compared gene expression between cells from each selected cell cluster versus all remaining cells from the other clusters. Marker genes were defined as significantly up-regulated genes in associated cell clusters with average natural-log fold change (avgLogFC) greater than 0.25 and Bonferroni adjusted p value less than 0.05, while top DEGs were also included down-regulated genes with avgLogFC less than −0.25.

#### GSEA hallmark enrichment analysis

For gene set enrichment analysis, all expressed genes were ranked in descending order by multiplying the negative log-p value (NLP) derived from DEG analysis by the sign of the avgLogFC between the two clusters. The resultant pre-ranked gene list was used as input into GSEA v4.0.3 Preranked analysis.^91^ The enrichment score is derived from a multivariate U score.^98^^,^^99^ Briefly, scores were calculated by averaging normalized expression levels for all the transcripts that were identified as maturation-associated DEGs, which were differentially expressed in any two adjacent stages of plasma cell maturation in Figure 1C or differentially expressed between early and late cell clusters and annotated in the selected HALLMARK pathways.^37^

#### Trajectory analysis

For the IgG dominant cell trajectory analysis, we used the slingshot method as it can detect the bifurcation, multifurcation, linear and tree-like differentiation topology.^89^ We ran slingshot r package v1.4.0 (*99*) with UMAP embeddings as input data, cell cluster ids as cell labels and cluster 1 (PB) as the starting point, and all other values using default settings. Although numerous methods have been developed for single cell trajectory imputation, strikingly, out of the 45 methods reviewed by this study,^100^ only three (PAGA and RaceID/StemID) can be used to detect disconnected graphs, but none of these releases are currently stable. We assumed that each lineage had its own progenitor population, so for the accuracy and stability of inference, we focused on dissecting the maturation paths only for IgG lineage cell populations which are likely to originate with the PB (cluster 1), whereas the progenitor for IgM remains unclear.

#### Transcription factor analysis

Transcription factors were combined from AnimalTFDB^101^ and known human transcription factors.^102^ For downstream analysis, we only focused on the TFs that were either identified as HVFs or markers of any one of cell clusters or differentially expressed in the comparison of any adjacent two clusters of PC maturation or between the early and late phases of PC maturation. Only those TFs expressed in at least 10% of any one of the BMPC subgroups were included in detection of cluster-specific TFs; and only those expressed in at least 20% of associated cell clusters and having avgLogFC greater than or equal to 0.25 between the two cell groups with the highest and lowest gene expression were selected for visualization. Averaging the expression of TFs by cluster id and defined maturation stage, each TF was assigned to the cell cluster or stage with the highest expression. For those TFs showing greater than or equal to 0.25 avgLogFC between assigned cell cluster and cell cluster with the second highest expression were labeled as cluster distinct. The same criterion was used to label stage-distinct TFs. Associations between pairs of TFs were evaluated using annotations in the STRING protein-protein interaction database^38^; only interactions that have a combined score greater than 500 and exist within the assigned cell cluster were retained for visualization.

#### Multicolor flow cytometry for experimental validation

MNC were isolated from 4 (Panel 1) and 2 (Panel 2) BM aspirate samples from heathy donors using a Ficoll density gradient and stained with the following anti-human antibodies: IgD-Brilliant Violet 480 (Cat. No. 566138; BD Biosciences), CD3-BUV737 (Cat. No. 612750; BD Biosciences), CD14-BUV737 (Cat. No. 612763; BD Biosciences), CD19-Spark NIR 685 (Cat. No. 302270; BioLegend), CD38-Brilliant Violet 785 (Cat. No. 303530; BioLegend), CD138-APC-R700 (Cat. No. 566050; BD Biosciences), CD27-Brilliant Violet 711 (Cat. No. 356430; BioLegend), BCMA-Brilliant Violet 421 (Cat. No. 357520; BioLegend), TACI-PE-Cy7 (Cat. No. 311908; BioLegend), OX40-Brilliant Violet 510 (Cat. No. 350026; BioLegend), GITR-Brilliant Violet 605 (Cat. No. 747664; BD Biosciences), and Zombie NIR Fixable Viability Kit (Cat. No. 423106; BioLegend). The anti-human antibodies that were used for staining BM MNC isolated from the patient with high donor-specific antibodies (DSA) awaiting kidney transplant in the ITN protocol (ClinicalTrials.gov Identifier: NCT04827979) include: IgD-BB700 (Cat. No. 566538; BD Biosciences), CD3-PE-Cy5 (Cat. No. 2363822; ThermoFisher), CD14-PE-Cy5 (Cat. No. 2319032; ThermoFisher), CD19-BUV395 (Cat. No. 740287; BD Biosciences), CD38-FITC (Cat. No. CYT-38F2-A; Cytognos), CD138-PE-Cy7 (Cat. No. 356514; BioLegend), CD27-BV605 (Cat. No. 562655; BD Biosciences), and Live/Dead (Cat. No. L34962; ThermoFisher). Cells were acquired on a Cytek Aurora spectral flow cytometer using Cytek SpectroFlo software (Cytek; the HD BM samples) or an LSR Fortessa X20 (special order research product with 5 lasers; BD Biosciences) and analyzed using FlowJo software (v10.8.1; with DownSample (v3.3.1) plugin; BD Biosciences).

#### Single cell VDJ sequencing (scVDJ-seq) and analysis

Cells were counted using a Bio-Rad TC10 cell counter and verified via hemocytometer. Cell numbers were adjusted to 1,000 cells per μl to allow for 10,000 single cells per sample loaded in the 10x Genomics Chromium device. The manufacturer’s standard protocol for Chromium Next GEM Single Cell 5′ Reagent Kit, v2 and Chromium Single Cell Human BCR Amplification kit was used to generate libraries. Libraries were sequenced on an Illumina NovaSeq (paired-end; 2 × 150 bp; read 1:26 cycles; i7 index: 8 cycles, i5 index: 0 cycles; read 2: 98 cycles) such that more than 70% saturation could be achieved with a sequence depth of 5,000 reads per cell for VDJ libraries.

Analysis of single cell VDJ data was conducted using Cellranger v3.1.0 via the 10x Genomics cloud interface and an in-house developed informatics pipeline for clonal clustering and SHM analysis.^69^ Fasta files from the Cellranger output were annotated with metadata and aligned to germline sequences using the IMGT/HighV-QUEST web portal.^103^ All data from IMGT/HighV-QUEST were retained through the process and were used for mutation calculations and alignment analyses. The definition of lineages/clones is consistent with previous publication.^69^ The frequency and distribution of somatic hypermutation were ascertained on the basis of non-gap mismatches of expressed sequences with the closest germline V_H_ sequence. Mutation frequencies were determined by calculation of the number of mutations in V regions relative to the number of bases in non-gap V regions. The ratio of replacement mutations to silent mutations were calculated for CDR and framework regions separately from the corresponding V_H_ areas. In sequences with non-zero replacement but zero silent mutations, the number of silent mutations was set to 1.^104^ Merging of gene expression and VDJ data sets and subsequent analysis was conducted using in-house developed pipelines in conjunction with the immcantation pipeline^105^ and Seurat. Circular visualization plots were created with Circos software v0.69-6.

#### Permutation test for mutation detection

In order to assess whether mutation numbers differ among clusters, we first randomly shuffled mutation frequency data in all the cells and grouped them using current cell clusters. We then performed ANOVA test and Tukey’s HSD (honest significant difference) consecutively. After repeating the previous steps one hundred times, we applied multiple comparison adjusted p values from the TukeyHSD test to calculate the p value for permutation test: P(permutation test) = (Number of permutation tests showing p value from TukeyHSD < p value obtained from running real data)/100.

## Data Availability

The raw and processed scRNA-seq and scVDJ-seq data generated during this study are publicly available at the Gene Expression Omnibus (GEO) GSE230705. The GEO accession number for these datasets is listed in the key resources table. The custom code used to process and visualize the data is deposited at Zenodo, and the accession DOI for the code is listed in the key resources table. Any additional information required to reanalyze the data reported in this work paper is available from the lead contact upon request.

## Supplemental information

Document S1. Figures S1–S13

Table S1. Cell cluster marker genes

Table S2. Summary of 205 differentially expressed TFs

Table S3. Comparison of top DEGs between 5 vs. 6 and 5 vs. 7

Table S4. Comparison of GSEA preranked results between 5 vs. 6 and 5 vs. 7

Table S5. HALLMARK_TNFA_SIGNALING_VIA_NFKB output results by running GSEA prerank analysis

Table S6. Tukey honestly significant difference *post hoc* test for TNF family gene expression between compared two clusters

Table S7. Tukey honestly significant difference *post hoc* test for the whole V (global), CDR, and FR regions mutation frequency among different isotypes

Document S2. Article plus supplemental information
